# Current methodologies in protein ubiquitination characterization: from ubiquitinated protein to ubiquitin chain architecture

**DOI:** 10.1186/s13578-022-00870-y

**Published:** 2022-08-12

**Authors:** Mingwei Sun, Xiaofei Zhang

**Affiliations:** 1 Basic Research Center, Bioland Laboratory, Guangzhou Regenerative Medicine and Health Guangdong Laboratory, Guangzhou, 510530 China; 2 Key Laboratory of Biological Targeting Diagnosis, Therapy and Rehabilitation of Guangdong Higher Education Institutes, The Fifth Affiliated Hospital of Guangzhou Medical University, Guangzhou, 510530 China; 3 CAS Key Laboratory of Regenerative Biology, Guangdong Provincial Key Laboratory of Stem Cell and Regenerative Medicine, GIBH-HKU Guangdong-Hong Kong Stem Cell and Regenerative Medicine Research Centre, Guangzhou Institutes of Biomedicine and Health, Chinese Academy of Sciences, Guangzhou, 510530 China

**Keywords:** Post-translational modification, Ubiquitination, Deubiquitination, Mass spectrometry

## Abstract

Ubiquitination is a versatile post-translational modification (PTM), which regulates diverse fundamental features of protein substrates, including stability, activity, and localization. Unsurprisingly, dysregulation of the complex interaction between ubiquitination and deubiquitination leads to many pathologies, such as cancer and neurodegenerative diseases. The versatility of ubiquitination is a result of the complexity of ubiquitin (Ub) conjugates, ranging from a single Ub monomer to Ub polymers with different length and linkage types. To further understand the molecular mechanism of ubiquitination signaling, innovative strategies are needed to characterize the ubiquitination sites, the linkage type, and the length of Ub chain. With advances in chemical biology tools, computational methodologies, and mass spectrometry, protein ubiquitination sites and their Ub chain architecture have been extensively revealed. The obtained information on protein ubiquitination helps to crack the molecular mechanism of ubiquitination in numerous pathologies. In this review, we summarize the recent advances in protein ubiquitination analysis to gain updated knowledge in this field. In addition, the current and future challenges and barriers are also reviewed and discussed.

## Introduction

**Fig. 1:**
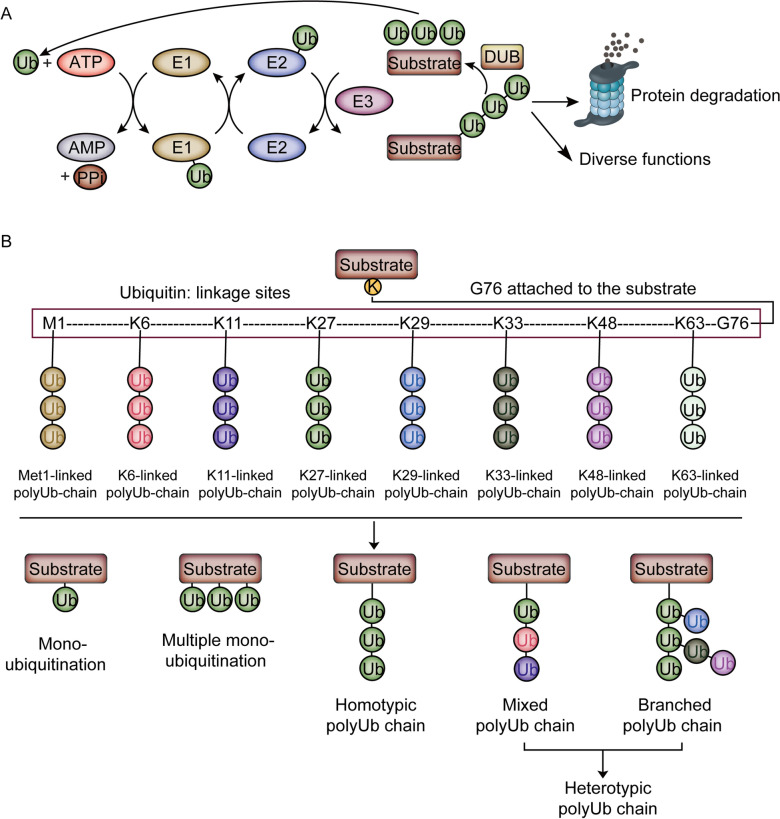
The cycle of ubiquitin signaling and ubiquitin proteoforms. **A** Schematic representation of the cycle of ubiquitin signaling. Ubiquitin monomers are activated by E1 ubiquitin-activating enzymes for E2 ubiquitin conjugation which works with or without E3 ubiquitin ligases to attach the activated ubiquitin to the target. The ubiquitin monomers are receded from the ubiquitinated substrates into ubiquitin pools by DUBs and reused. **B** Schematic diagram of the various forms of ubiquitylation. A protein can be modified by mono-, multi-mono- or polyubiquitin. Polyubiquitin is categorized into three types: homotypic polyUb chain, mixed polyUb chain, and branched polyUb chain. Ubiquitin chains are colored according to linkage type, and different color represents different linkage types

The covalent attachment of ubiquitin (Ub) to protein substrate (ubiquitination) is an important post-translational modification (PTM) that regulates diverse cellular functions [1–4]. Ub is a small and highly conserved 76-residue protein in eukaryotes. The C-terminal glycine of Ub (G76) is covalently attached to its substrate protein, which is regulated in a cascade order of E1 Ub-activating enzymes, E2 Ub-conjugating enzymes, and E3 Ub ligases (Fig. 1A) [5, 6]. Ub is reversibly removed from the substrate through Ub hydrolases known as a family of proteins named deubiquitinases (DUBs) [7]. Maintenance of cellular ubiquitination homeostasis is fulfilled by the orchestrated interplay of 2 E1 enzymes, ~ 40 E2 enzymes, more than 1000 E3 ligases, and approximately 100 DUBs encoded by the human genome [8–10]. Aberrance of ubiquitin-related enzymes’ activity leads to the dysregulation of protein ubiquitination, promoting the pathogenesis of numerous diseases, such as cancer, neurodegenerative diseases, and so on [11–14].

Ub can be covalently linked to one or more accessible modification residues of the substrate, resulting in the formation of mono-ubiquitination and multiple mono-ubiquitination, respectively (Fig. 1B). In addition, Ub containing one N-terminal methionine residue (M1) and seven lysine residues (K6, K11, K27, K29, K33, K48, K63), provides 8 free –NH2 groups as linkage sites for conjugating with the C-terminus of distal Ub molecule, resulting in different polyUb chains. polyUb with the same linkage type is named homotypic chains, whereas heterotypic chains contain mixed and branched linkage types [15, 16]. In addition, other PTMs, such as phosphorylation and acetylation, occur on the Ub, which further complicates the ubiquitination signaling [17]. Protein ubiquitination is recognized by different effector proteins containing distinct Ub binding domains (UBDs) to result in diverse outcomes [18–21]. K48-linked ubiquitin chains, which are the most abundant Ub linkage in cells, target substrate protein to the 26S proteasome for degradation, and removal of K48-linked chains from the substrate by DUBs prevents degradation [22–25]. K63-linked chains regulate protein–protein interactions to activate protein kinases during activation of the NF-κB pathway and autophagy [26, 27]. In comparison, the functions of atypical chain types, including mono-ubiquitination, multi-ubiquitination, K6-/K11-/K27-/K29-/K33-linked ubiquitin chains, M1-linked linear ubiquitin chain, are less well defined. We recently reviewed the advances in atypical ubiquitination, showing that identification and enrichment of atypical Ub chains remains a critical challenge [8].

It is a critical challenge to characterize protein ubiquitination. First, the stoichiometry of protein ubiquitination is very low under normal physiological conditions which increases the difficulty to identify the ubiquitinated substrates. Second, Ub could modify the substrates at one or several lysine residues simultaneously, which significantly increases the difficulty to localize the ubiquitination sites using traditional methods. Third, Ub itself can serve as a substrate, thereby resulting in the complexity of Ub chains which vary in length, linkage, and overall architecture [28]. Therefore, to better understand the function of protein ubiquitination, novel approaches or strategies are required to globally paint the picture of protein ubiquitination, including the substrates, modified sites, Ub linkages, and the chain’s architecture. In this review, we summarize the approaches or strategies that have been developed for profiling the ubiquitinated proteins, protein ubiquitination sites as well as Ub linkages, and its chain’s architecture.

## Insights into ubiquitination at the protein level

Traditionally, protein ubiquitination was identified through biochemical approaches. In conventional approaches, immunoblotting was used to test the level of putative substrate ubiquitination through anti-Ub antibodies [29]. If the immunoblotting result suggested ubiquitination on the substrates, the putative ubiquitinated lysine was further mutated and analyzed by immunoblotting to evaluate whether the mutated lysine was ubiquitinated or not [30, 31]. Using this kind of strategy, Ortiz et al. found that the ubiquitination level of Merkel cell polyomavirus large tumor (LT) antigen was significantly reduced when K585 was substituted with R585, indicating that K585 is the ubiquitination site [32]. The conventional approaches are the most widely used ones for the detection and validation of ubiquitination for a single protein. However, the immunoblotting approach is a time-consuming and low-throughput analytical method, limiting its application in protein ubiquitination profiling.

**Fig. 2:**
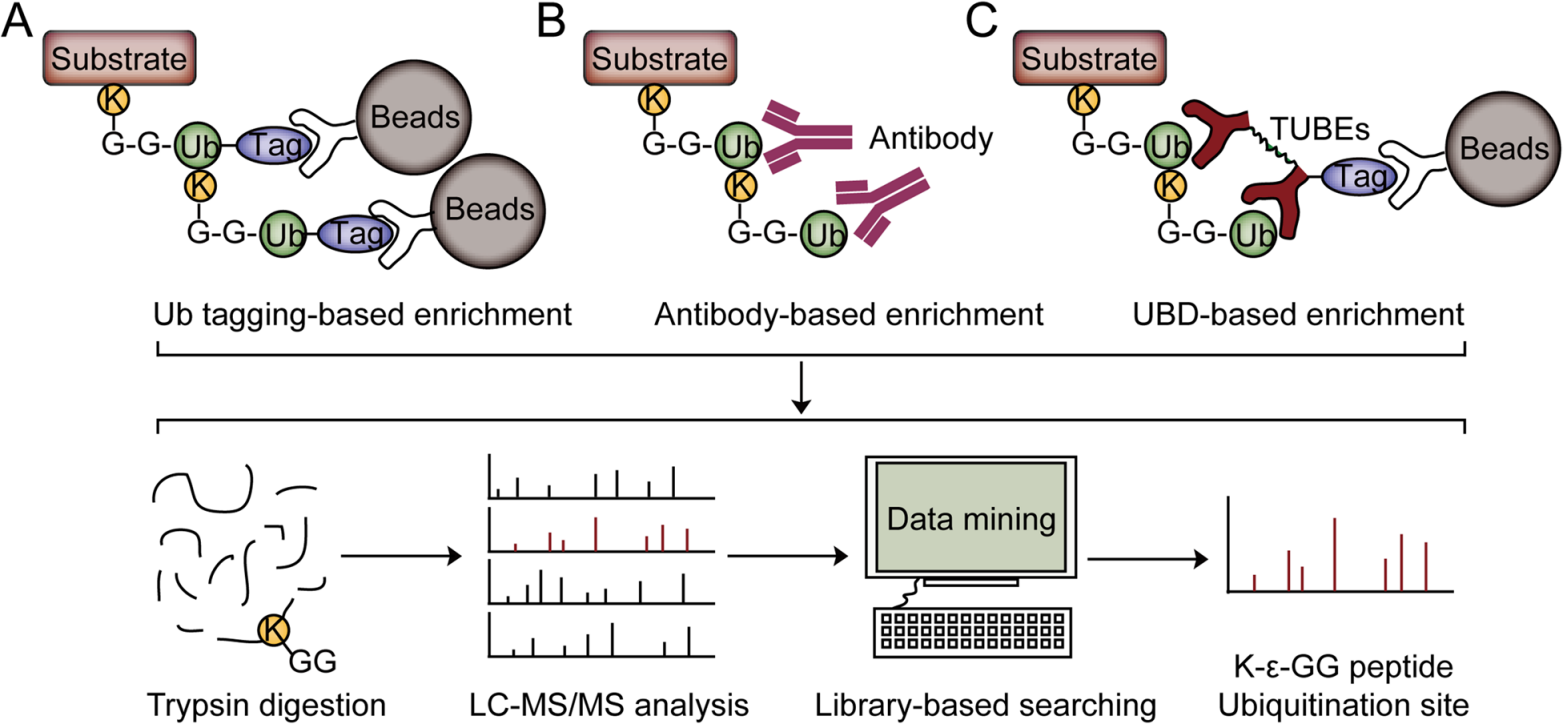
MS-based approaches to identifying the ubiquitination sites followed by enrichment of ubiquitinated proteins. **A** Purification of ubiquitylated proteins using tagged ubiquitin. **B** Purification of ubiquitylated proteins using Ub antibody. **C** Purification of ubiquitylated proteins using UBDs. The ubiquitinated proteins are first purified by the corresponding materials and digested by trypsin. Then, the whole tryptic peptides are analyzed by LC–MS/MS, resulting in the ubiquitination identification from the complicated peptide pool

With advances in mass spectrometry (MS), researchers are increasingly turning their attention to profile the ubiquitination by MS-based proteomics. To increase the identification sensitivity of protein ubiquitination, it is important to enrich ubiquitinated substrates from the whole cell lysates to avoid interference from non-ubiquitinated ones. Here, we will discuss the approaches to enriching ubiquitinated proteins and identifying the ubiquitination sites by MS-based proteomics (Fig. 2).

### Insights into ubiquitinated proteins using Ub tagging-based approaches

To profile protein ubiquitination in a high-throughput manner, the ubiquitinated proteins were enriched after the expression of Ub tags in living cells. There are two kinds of tags used for purifying ubiquitination substrates, termed epitope tags and protein/domain tags. The epitope tags are usually small peptides, including Flag, HA, V5, Myc, Strep, and His. And protein/domain tags contain GST, MBP, SUMO, CBP, Halo, Nus A, and FATT [33]. His tag and Strep-tag are the most commonly used affinity tags in protein ubiquitination profiling [1, 8].

After expressing the Ub containing affinity tag, the ubiquitinated substrates are covalently labeled by the affinity tag. Therefore, ubiquitinated proteins can be enriched using commercially available resins (Ni-NTA for His tag and Strep-Tactin for Strep-tag) and identified by MS-based proteomics. In 2003, Peng et al. firstly reported a proteomic approach to enriching, recovering, and identifying protein ubiquitination from *Saccharomyces cerevisiae* through expressing 6× His-tagged Ub. After purification and tryptic digestion of ubiquitinated proteins, the ubiquitination sites were determined by MS analysis through the identification of 114.04 Da mass shift on the modified lysine residues. Finally, Peng et al. identified 110 ubiquitination sites on 72 proteins [34]. Similarly, Akimov et al*.* reported a stable tagged Ub exchange (StUbEx) cellular system in which endogenous Ub was replaced with a His-tagged Ub [35]. Eventually, 277 unique ubiquitination sites on 189 proteins were identified in HeLa cells. In addition to His tag, Strep-tag is another affinity tag used for the purification of ubiquitinated proteins, which can bind strongly to Strep-Tactin. By constructing a cell line stably expressing Strep-tagged Ub, Danielsen et al. identified 753 lysine ubiquitylation sites on 471 proteins in U2OS and HEK293T cells [36]. In conclusion, Ub tagging-based approach is an easy and friendly method to screen the ubiquitinated substrates in cells with a relatively low-cost feature.

Although Ub tagging-based approaches enable protein ubiquitination profiling, there are some disadvantages to these approaches. First, histidine-rich, and endogenously biotinylated proteins can be co-purified using Ni-NTA agarose and Strep-Tactin-based resins, respectively. Therefore, a large number of peptides derived from non-ubiquitinated proteins impair the identification sensitivity of protein ubiquitination. Second, tagged Ub may change the structure of Ub, which cannot completely mimic the endogenous Ub. Therefore, artifacts might be generated using Ub tagging-based approaches. Third, the identification efficiency of this method is relatively low. In addition, expressing tagged Ub in animal or patient tissues is infeasible, limiting the application of Ub tagging-based approaches in tissues. Therefore, approaches, which enable enriching the endogenously ubiquitinated proteins, are attractive.

### Insights into ubiquitinated proteins using Ub antibody-based approaches

To profile endogenously ubiquitinated substrates, several types of anti-Ub antibodies, such as P4D1, and FK1/FK2 that recognized all Ub linkages, were developed to enrich and detect ubiquitinated substrates. For example, Denis et al. used FK2 affinity chromatography to enrich ubiquitinated proteins from human MCF-7 breast cancer cells and identified 96 ubiquitination sites by MS analysis [37]. In addition to non-specific Ub antibodies, linkage-specific antibodies are available for enrichment of ubiquitinated proteins with specific chain linkages (M1-/K11-/K27-/K48-/K63-linkage specific antibodies) [1, 8, 38, 39]. Nakayama et al*.* generated a novel antibody that specifically recognized K48-linked polyUb chains and found that K48-linked polyubiquitination of tau proteins was abnormally accumulated in Alzheimer’s disease [40]. This antibody-based approach is successfully applied to characterize protein ubiquitination from animal tissues or clinical samples without the need for genetic manipulation [1]. Although the antibody-based approaches can identify protein ubiquitination under physiological conditions and get insights into the chain-linkage information by using linkage-specific antibodies, the high cost of antibodies and non-specifically binding of potential proteins are the disadvantages.

### Insights into ubiquitinated proteins using UBD-based approaches

Proteins containing UBDs (some E3 Ub ligases, DUBs, and Ub receptors) recognize Ub linkage in general or selectively, which can be utilized to bind and enrich endogenously ubiquitinated proteins [41–44]. Single UBD was firstly used to enrich ubiquitylated proteins, but the low affinity of single UBD limited its application in the purification of ubiquitinated proteins. Tandem-repeated Ub-binding entities (TUBEs) exhibited significantly higher affinity (low nanomolar) than a single UBD domain and stabilized ubiquitylated proteins in cell extracts [45]. Therefore, TUBEs have emerged as promising methods to enrich ubiquitinated proteins [46–49].

By the combination of 1D SDS-PAGE and MS, Mata et al. reported an approach based on GST-TUBEs to identify ubiquitinated proteins in both *Plasmodium* and its infected red blood cells, resulting in identifying 23 ubiquitylated peptides on 12 proteins [47]. To avoid degradation of ubiquitinated proteins during sample preparation and to eliminate the interference of the peptides derived from Ub and polyUb chains during MS analysis, Yoshida et al*.* developed a trypsin-resistant TUBEs (TR-TUBEs) approach, allowing detection of the specific activity of the Ub ligase and enrichment of its regulated ubiquitinated substrates [50]. To enrich various kinds of Ub linkages simultaneously, tandem hybrid UBD (ThUBD)-based approaches were developed to enrich the ubiquitinated proteins from cell lysates [51–53]. Using ThUBD-based profiling with MS, Gao et al*.* identified 362 and 1125 ubiquitinated proteins in yeast and mammalian cells, respectively [51]. In addition, TUBEs for specific chain linkages including K63 TUBEs, K48 TUBEs, and M1 (Linear) TUBEs, have also been developed [43, 45, 49, 54]. For example, Back et al*.* utilized the K63 TUBE system to enrich K63 ubiquitinated proteins followed by MS analysis and quantified > 1100 K63 ubiquitination sites in H_2_O_2_ treated yeast [55].

While TUBEs have several advantages over other technologies, there are limitations. First, TUBEs enrich monoubiquitylated proteins with much lower affinity than polyubiquitylated proteins. Since approximately 50% of protein ubiquitination exists in the monoubiquitylated state [49], TUBEs-based approaches will miss a large part of monoubiquitylated substrates. Second, plenty of non-targeted peptides will be derived from TUBEs and the polyUb chains after tryptic digestion, which may reduce the identification sensitivity of ubiquitylated substrates.

### Other approaches to getting insights into ubiquitinated proteins

In addition to the strategies based on Ub tagging, Ub antibody, and UBD, other approaches based on novel binding proteins capable of enriching ubiquitinated proteins, such as aptamers and affimers, were developed [49]. For example, Michel et al*.* utilized K6- and K33-linkage-specific “affimer” reagents to enrich the Ub conjugates and performed the absolute quantification (AQUA) analysis by MS which provided information for the precise determination of Ub chains [56]. These tools can also be applied in MS studies to identify specific targets of these modifications.

## Insights into ubiquitinated proteins at the peptide level

**Fig. 3:**
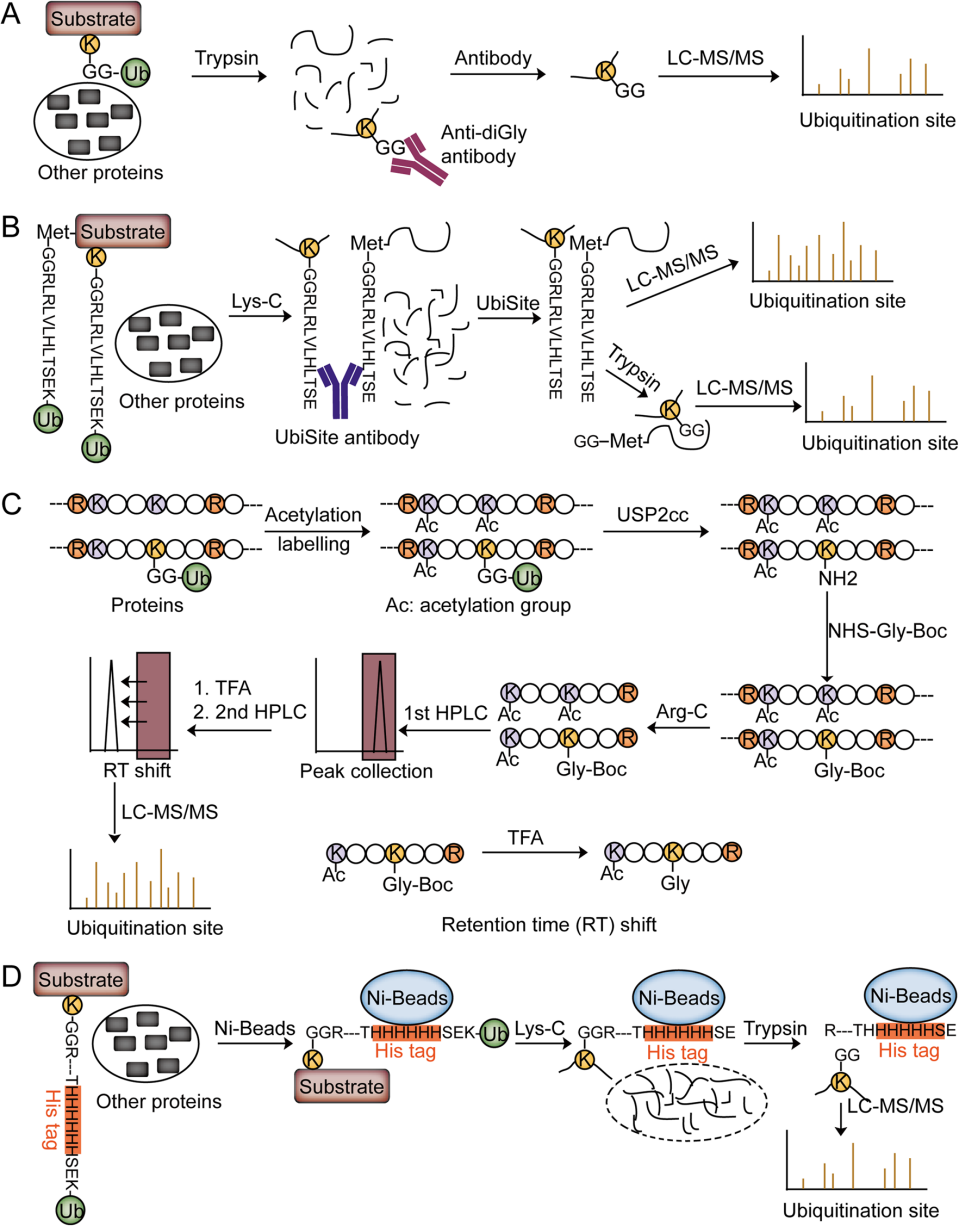
Approaches to identifying ubiquitination sites followed by the enrichment of ubiquitinated peptides. **A** Schematic workflow of anti-diGly antibody-based approach. The whole cell lysates are directly digested with trypsin. Then, the diGly-modified peptides are further enriched by the anti-diGly antibody for LC–MS/MS analysis. **B** Schematic workflow of UbiSite antibody-based approach. Ubiquitinated proteins are first digested with Lys-C. Then, the ubiquitinated peptides containing the ESTLHLVLRLRGG sequence are enriched by the UbiSite antibody. The enriched peptides are directly analyzed or analyzed after tryptic digestion by LC–MS/MS. **C** Schematic workflow of Ub-COFRADIC approach. After labeling the lysine ε-amine by acetylation, the ubiquitin moieties are hydrolyzed from the substrates using USP2cc to reveal a free primary amine on the ubiquitinated lysine. After Gly-Boc labeling and Arg-C digestion, the peptides are separated by a first RP-HPLC into some fractions. Each fraction is acidized by TFA to remove the Boc group. The retention time of Gly-Boc-modified peptides is different from the retention time of Gly-modified peptides. After secondary RP-HPLC runs, the peaks with a retention time shift are collected and analyzed by LC–MS/MS, resulting in ubiquitination site identification. **D** Schematic workflow of StUbEx PLUS approach. Ubiquitinated proteins are purified by Ni-beads and digested with Lys-C to specifically cleave after Lys residues (K). Therefore, the non-ubiquitinated peptides are released from the Ni-beads and the ubiquitinated peptides are still attached to the Ni-beads. By specifically cleaving after Arg residues (R), the ubiquitinated peptides containing diGly remnants are purified from the Ni-beads and identified by LC–MS/MS analysis

Most of the above approaches to enriching protein ubiquitylation are at the protein level. Upon digested by various protease enzymes, the majority of peptides are non-ubiquitinated ones derived from the ubiquitylated proteins or interacting proteins or Ub linkages, which significantly obscure the identification of ubiquitinated peptides. To overcome these limitations, it is vital to develop an approach that directly enriches ubiquitylated peptides from the digested peptide pool. The enrichment of ubiquitinated peptides containing Ub remnant can be achieved by antibody-based or antibody-free approaches that specifically recognize or separate the lysine peptides containing the ubiquitination sites. By analyzing the enriched-ubiquitinated peptides through MS-based proteomics, thousands of ubiquitination sites will be identified. Nowadays, several strategies have been reported to enrich the ubiquitinated peptides at the peptide level (Fig. 3).

### Anti-diGly antibody-based approach to profiling ubiquitination sites

Because the C-terminal sequence of the Ub is KESTLHLVLRLRGG and the last glycine is conjugated to lysine residues on target proteins, after tryptic digestion, a diglycine (diGly) remnant was left on the substrate lysine residue (K-ε-GG). By analyzing diGly remnant induced mass shift, ubiquitination sites can be well identified and quantified by MS. An essential breakthrough in MS-based ubiquitination analysis was achieved with the development of an antibody that specifically recognizes peptides containing diGly remnant. In 2010, Xu et al*.* published a groundbreaking work to globally profile protein ubiquitination in a human cell line (Fig. 3A). They identified 374 ubiquitination sites on 236 proteins from HEK293 cells [57]. Since then, multiple techniques in sample preparation and MS analysis were optimized to improve the identification depth of ubiquitylation sites. By the combination of anti-K-ε-GG antibody enrichment and pre-fractionation, another two groups demonstrated the feasibility of this combination by identifying 11,054 and 19,000 ubiquitylation sites in human cells [58, 59]. To reduce the sample amounts and the trypsin missed cleavages, Casanovas et al*.* developed a large-scale filter-aided sample preparation (LFASP) method and identified more than 10,000 ubiquitination sites using 1.2 mg proteins in human cell lines [60]. To improve the identification efficiency of protein ubiquitylation, Xu et al*.* developed a strategy by digesting ubiquitylated proteins with both trypsin and Ac-LysargiNase [61]. Through this strategy, the identification of ubiquitination sites was consequently increased by 30% to 50%. More recently, Wagner et al*.* developed an Orbitrap-based data-independent acquisition (DIA) method combining K-ε-GG enrichment and optimization with a spectral library containing more than 90,000 K-ε-GG peptides. This approach identified 35,000 K-ε-GG peptides in a single run of proteasome inhibitor-treated cells, significantly improving the sensitivity of protein ubiquitination identification [62]. In summary, with the optimizations in sample preparation, mass spectrometry, data acquisition mode, and data analysis algorithm, the sensitivity of ubiquitination analysis using the Anti-diGly antibody-based proteomics approach is significantly improved, enhancing the comprehensive mapping of ubiquitination signal at large scale and the understanding of ubiquitination function in associated diseases. Therefore, this method is now the most widely used approach for ubiquitination analysis.

Although the current ubiquitination enrichment strategies provide proteome-wide profiling of protein ubiquitylation sites, anti-K-ε-GG antibody-based proteomic strategies exhibit several issues. One of the issues is related to protein digestion. If trypsin does not cleave the arginine residues of Ub completely (the C-terminal sequence of Ub: KESTLHLVLRLRGG), LRGG modification on Lys may occur on the substrates. However, the anti-K-ε-GG antibody doesn’t recognize the LRGG group, resulting in the missing identification of some ubiquitylated peptides. Another issue relates to the proteolytic digestion of other Ub-like proteins, such as NEDD8 and ISG15. These Ub-like proteins generate the same diGly remnant on modified lysine residues, which makes ubiquitination, Neddylation, and ISGylation indistinguishable based on the tryptic remnant. The third issue is chemical artifacts induced in cysteine alkylation using iodoacetamide (IAA). IAA reacts with lysine, generating a 2-acetamidoacetamide artifact with identical mass to the diGly remnant which will interfere with ubiquitination identification by MS [63]. This IAA-induced artifact can now be avoided by using less reactive chloroacetamide or by lowering the reaction temperature and concentration of iodoacetamide [64]. In addition, the profiling of K-ε-GG peptides using antibodies may exhibit slight peptide sequence bias [65].

### UbiSite antibody-based approach to profiling ubiquitination sites

To overcome some drawbacks associated with the diGly approach, Akimov et al*.* recently generated an antibody, UbiSite, which recognized the C-terminal 13 amino acids (ESTLHLVLRLRGG) of Ub after Lys-C digestion (Fig. 3B). By combining UbiSite-based enrichment and pre-fractionation, the authors identified over 63,000 unique ubiquitination sites on 9200 proteins in human Hep-G2 and Jurkat cell lines after proteasomal inhibitor treatment by MS analysis [66]. In addition, 104 N-terminal ubiquitination sites were identified in their results which were ignored by the diGly approach. As the only available tool with the ability to enrich peptides from protein N-terminal ubiquitination, the UbiSite antibody-based approach might be a useful approach to studying the function and regulation of N-terminal ubiquitination [67].

Firstly, due to the specific C-terminal sequence (ESTLHLVLRLRGG) of Ub, the UbiSite antibody significantly improves specificity toward ubiquitinated peptides by reducing the non-specific enrichment of Ub-like proteins such as NEDD8 and ISG15. Secondly, UbiSite enables enrichment of protein N-terminal ubiquitination, which advances the understanding of protein N-terminal ubiquitination. Hoverer, the sequence ESTLHLVLRLRGG is quite long on the ubiquitinated peptides, resulting in more extra fragments during MS/MS analysis, which will impair the identification efficiency of protein ubiquitination using Lys-C digestion only [45]. Therefore, extra enzymatic digestion will benefit the protein profiling for the UbiSite approach [45].

### Insights into N-terminal ubiquitination using the antibody toolkit

Conjugation of Ub to lysine ε-amino group is the most common type of ubiquitination. Besides, the α-amino group of protein N-terminus has also been identified as non-canonical ubiquitinated targets [68, 69]. However, current approaches, except the UbiSite approach, mainly focus on lysine ubiquitination profiling, there are limited approaches to analyzing the N-terminal ubiquitination, which hampers the functional study of protein N-terminal ubiquitination. To identify protein N-terminal ubiquitination, three approaches for profiling protein N-terminal ubiquitination, anti-GGX mAbs based approach, UbiSite based approach, and StUbEx PLUS approach, were reported [66, 70, 71]. For example, Davies et al*.* generated four monoclonal antibodies (anti-GGX mAbs) that selectively recognize tryptic peptides with an N-terminal diGly remnant rather than the K-ε-GG group, realizing the specific enrichment and identification of protein N-terminal ubiquitination [70]. UBE2W is the only E2 Ub conjugating enzyme reported to regulate the protein N-terminal ubiquitination. The authors used anti-GGX mAbs to enrich and analyze UBE2W regulated N-terminal ubiquitination events [72]. They identified 152 unique N-terminal ubiquitination sites derived from 109 endogenous proteins. Of the 152 unique N-terminal ubiquitination sites, 32 sites are reported as the potential substrates of UBE2W, demonstrating that the anti-GGX mAbs-based approach is qualified for protein N-terminal ubiquitination profiling.

Among the three methods for analyzing N-terminal ubiquitination, the anti-GGX mAbs-based approach is designed for selectively enriching tryptic peptides with an N-terminal diglycine remnant rather than a diglycine remnant on lysine. However, the UbiSite antibody specifically recognizes the ubiquitin 13-residue remnant on N-terminus and Lys residue after Lys-C digestion. Different antibodies show some bias toward some preferent sequences. In addition, StUbEx PLUS (see below) is an antibody-free approach that is different from the above antibody-based approaches, showing no bias toward lysine and N-terminal ubiquitination. That’s why only a small part of N-terminal ubiquitination sites identified by the anti-GGX mAbs-based approach were overlapped with the ubiquitination sites identified by UbiSite and StUbEx PLUS approaches [66, 70, 71]. This result nicely highlights the limitations and complementary of the current approaches.

### Antibody-free approaches to profiling ubiquitination sites

Antibody-based strategies are the most widely used approaches to systematically profiling protein ubiquitination at the peptide level. However, antibody-based approaches to profiling protein ubiquitination entail some shortcomings such as (a) the bias toward the amino acid sequence surrounding the ubiquitination sites and (b) expensive antibodies limit the widespread application. As an alternative to antibody-based approaches, Gevaert et al*.* reported an antibody-free approach, termed Ub COmbined FRActional DIagonal Chromatography (COFRADIC) (Fig. 3C), for enriching and identifying protein ubiquitination at the peptide level [73, 74]. In brief, all primary amino groups were blocked by acetylation at the protein level, followed by USP2 catalytic core domain (USP2cc) incubation to hydrolysis Ub from the ubiquitinated proteins and reintroduce of the free ε-amine groups at the ubiquitination sites. To the free ε-amines, a glycine linked to a hydrophobic tert-butyloxycarbonyl (BOC) group was attached, which was further used to enrich the peptides via two reverse-phase HPLC (RP-HPLC) runs before and after TFA-based removal of BOC groups. Gevaert et al*.* used this approach to profiling protein ubiquitination in native human Jurkat cell lysate and in *Arabidopsis thaliana*, resulting in the identification of over 7500 endogenous ubiquitination sites on 3300 different proteins and 3009 endogenous ubiquitination sites on 1607 proteins, respectively.

Another reported antibody-free approach is StUbEx PLUS (Fig. 3D). Based on the Stable Tagged Ub Exchange (StUbEx) strategy which was used to enrich ubiquitinated proteins, Akimov et al*.* modified StUbEx strategy, StUbEx PLUS, to specifically enriched the ubiquitinated peptides [71]. The authors built a StUbEx PLUS system to insert His-tag between serine 65 and threonine 66 in the recombinant Ub. After proteolytic digestion with specific lysine cleavage enzymes, the tag was still attached to the ubiquitination sites and enriched by Ni-NTA beads. Since Ub-like protein doesn’t carry His-tag, the interference from Neddylation and ISGylation were also avoided here. Using the StUbEx PLUS strategy, 41,589 unique ubiquitination sites on 7762 proteins were identified in U-2 OS cells.

The antibody-free approach showed a powerful ability to enrich and profile protein ubiquitination, easier handling and cheaper antibody-free approaches for protein ubiquitination profiling are very much needed to overcome the drawbacks of antibody-based approaches. Recently, we proposed an antibody-free approach, termed AFUP, to profiling protein ubiquitination by selectively clicking the ubiquitinated lysine, resulting in 7103 ubiquitination site identification with high confidence in 5 mg HeLa lysates (in preparation).

Antibody-free-based strategies play a vital role in the studies of protein ubiquitination, which can overcome some drawbacks, for example, bias toward to sequence surrounding the ubiquitination sites and high cost. Even though antibody-free approaches enable global identification of protein ubiquitination, there still exist some shortcomings. First, the dataset identified by antibody-free approaches is quite smaller than the dataset identified by antibody-based approaches, suggesting that more effective antibody-free approaches are needed to expand the depth of the protein ubiquitination pool. Second, the Ub-COFRADIC approach is complicated and time-consuming. Third, StUbEx PLUS or other approaches like StUbEx PLUS require a tagged Ub which changes the structure of Ub, introducing some artifacts and limiting its application in animal or patient tissues. Therefore, an antibody-free approach, with high throughput, convenient and effective identification of protein ubiquitination, is urgently needed.

## Insights into the ubiquitinated proteins at the level of architecture

**Fig. 4:**
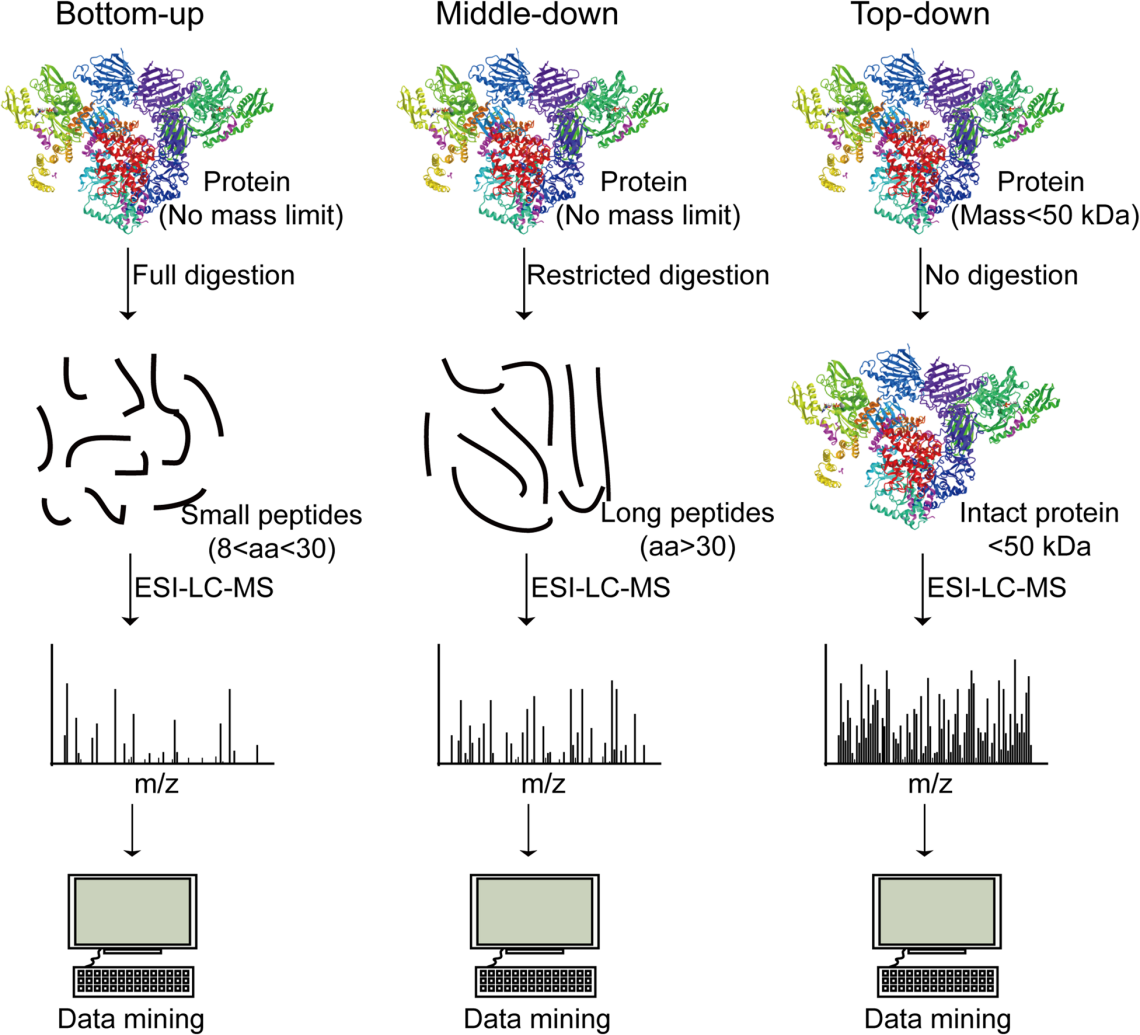
Schematic workflow of bottom-up proteomics, middle-down proteomics, and top-down proteomics. The protein structure is downloaded from National Center for Biotechnology Information (MMDB ID: 209664, PDB ID: 7KW7) [104].

**Fig. 5:**
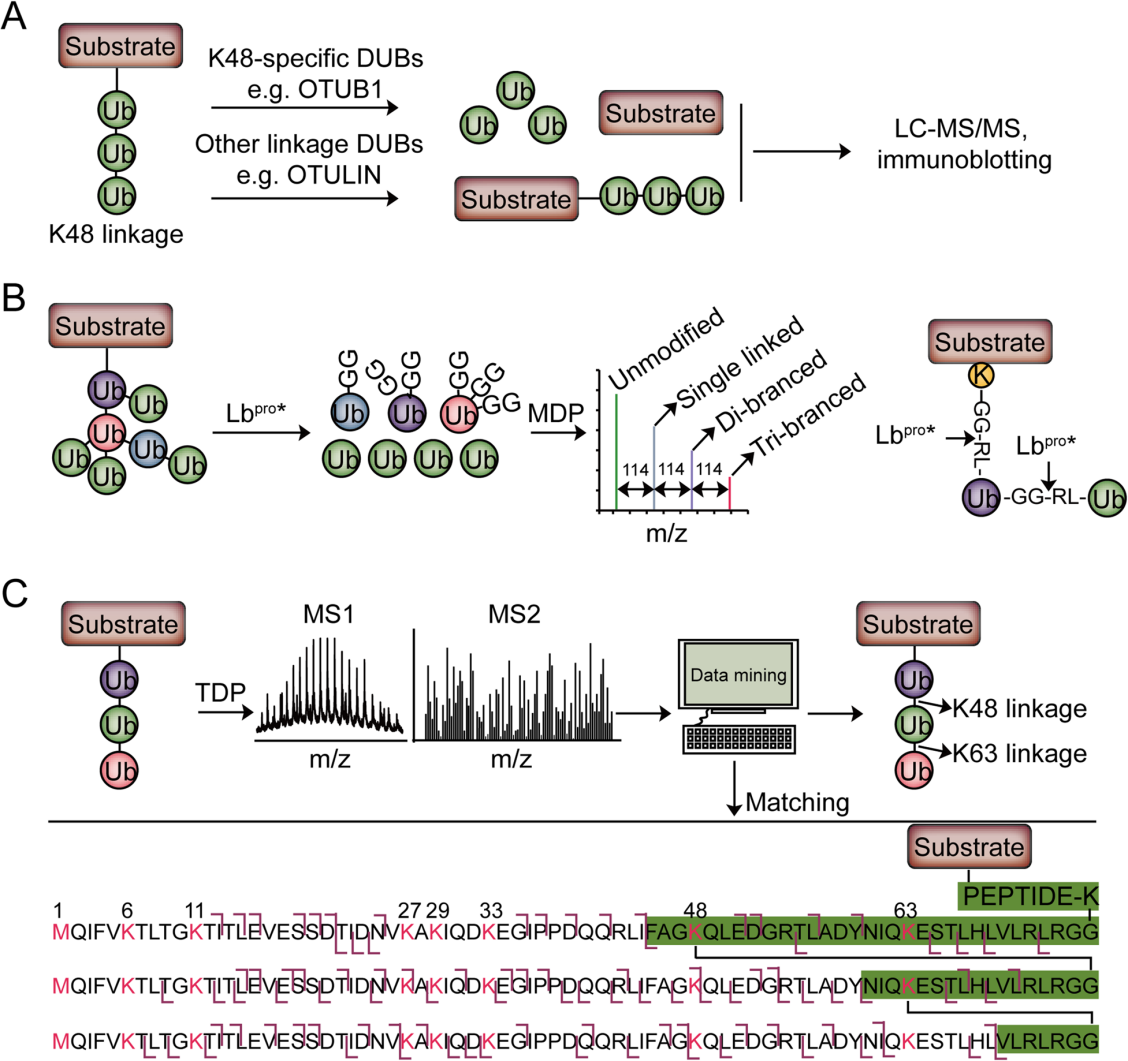
Approaches to getting insights into the architecture of Ub chains. **A** Schematic diagram of the BUP strategy of UbiCRest to characterize the substrate ubiquitin chain type. **B** Schematic diagram of the MDP strategy of Ub-clipping to characterize the substrate ubiquitin architecture. **C** Schematic diagram of TDP strategy to characterize the substrate ubiquitin architecture

Ub chains with distinct topologies regulate the protein stability, protein–protein interaction, or protein localization in eukaryotic cells, and thus play vital roles in multifunctional signals [75]. The approaches discussed above mainly reveal the ubiquitinated substrates and ubiquitination sites, which are not able to reveal the Ub chain architecture. There are several possibilities to detect the architectures of Ub chains. For example, linkage-specific antibodies which specifically recognize Ub linkages are used to identify the distinct topologies of Ub chains [8]. Currently, MS-based proteomics is categorized into bottom-up proteomics (BUP), middle-down proteomics (MDP), and top-down proteomics (TDP) based on the analytes (Fig. 4). Bottom-up is a traditional strategy of digesting proteins into small peptides for LC–MS analysis with high throughput. However, complete digestion leads to the inability to distinguish the protein isoforms. Middle-down is a restricted digestion proteomics strategy to generate longer peptides for LC–MS analysis, resulting in analyzing a wider range of peptide fragments. Compared with bottom-up proteomics and middle-down proteomics, Top-down proteomics strategy does not need digestion, but directly analyzes the intact protein by LC–MS to gain a comprehensive characterization of the analyzed protein. In this section, we mainly discuss the MS-based approaches to mapping the topologies of Ub chains (Fig. 5).

BUP is the conventional approach to analysis. A disadvantage of the BUP strategy is the loss of architectural information on polyUb or branched chains upon trypsin digestion. Therefore, whole-cell K-ε-GG analysis does not reveal the topological information of Ub chain attached to the substrates. To reveal the information of Ub chains, linkage-specific antibodies, Affimers, or binding domains are firstly used to enrich a specific chain type and the BUP strategy is further used to identify the linkage-specific substrates [38, 39, 56, 76]. There are several reviews that summarize the application of linkage-specific chain enrichment strategies to study the structure topologies of polyUb chains [28, 49, 77–79]. Mapping the topologies of Ub chains requires the detection of Ub molecules modified by other Ub molecules. Ohtake et al. used a mutated Ub in which the arginine 54 was replaced by an alanine. This mutant enables the discrimination between branched K48/K63 linkages and unbranched linkages [80]. The authors revealed that the Ub chain branched at K48 and K63 regulated nuclear factor κb (NF-κB) signaling. Ub chain restriction (UbiCRest) is another approach to analyzing Ub chain architecture (Fig. 5A), in which substrates (ubiquitinated proteins or polyUb chains) are treated with a panel of linkage-specific DUBs in parallel reactions [81]. Several issues should be considered when using the UbiCRest approach. First, some DUBs may be non-specific for unexpected Ub chains, leading to cross specificity. Second, many DUBs can’t hydrolyze long (n > 4) chains from the substrates [82]. Third, simple UbiCRest is unable for heterotypic chain analysis [83–85]. In addition to the methods described above, absolute quantification of ubiquitin (Ub-AQUA) by MS is a standard way to detect the multiformity of ubiquitin linkages that are covalently attached to the protein substrate. By synthesizing isotope-labeled internal standard-tryptic peptides corresponding to mono-ubiquitin and poly-ubiquitin chains bound to cyclin B1, Kirkpatrick et al*.* revealed that cyclin B1 was modified by complex ubiquitin chain architecture linked through Lys63, Lys11 and Lys48 [86]. By the combination of affinity chromatography and protein standard absolute quantification (PSAQ) mass spectrometry, Kaiser et al*.* developed a strategy, termed ubiquitin-PSAQ, to quantify cellular concentrations of ubiquitin species by spiking stable isotope-labeled free ubiquitin and ubiquitin conjugates into the lysates [87]. The authors used ubiquitin-PSAQ to measure the concentrations of ubiquitin types in both cell lines as well as mouse and human brain tissue and found that the concentrations of different ubiquitin types varied significantly in different samples. Therefore, BUD-based Ub-AQUA strategies will play vital roles in determining the ubiquitin topology of specific substrates and the concentrations of ubiquitin types of the whole proteome.

As an alternative, the MDP approach has been the most widely used in determining branched chains. Utilizing limited tryptic digestion, the MDP strategy has been applied in determining the abundance of branched Ub chains and detecting the specific linkages, such as K6/K48 and K29/K48 linkages [88–91]. Considering the high activity of trypsin towards Lys and Arg residues, it is difficult to control the process of tryptic digestion in the MDP strategy. Ub-clipping is a kind of MDP strategy (Fig. 5B), which uses an engineered viral protease, Lb^pro^*. Lb^pro^* is created by mutating the 102 Leu to Trp of Lb^pro^, a foot and mouth disease leader protease, to enable the preferable cleavage towards all types of diubiquitin. The cleavage of Lb^pro^* happens after Arg74 of Ub and leaves the signature C-terminal diGly attached to modified residue [92]. Swatek et al. used Ub-clipping to quantify branch-point Ub and surprisingly found that about 10–20% of Ub chains seemed to exist as branched type. The authors also showed that PINK1/PARKIN-mediated mitophagy predominantly exploited mono- and short-chain polyUb [93].

Compared to BUP and MDP, TDP may be an ideal platform for the analysis of proteomes bearing Ub chains of different lengths, linkages, and architectures (Fig. 5C) [28]. The major challenge of TDP is that its gas-phase dissociation produces overlapping and low signal-to-noise (S/N) fragments with increasing molecular weight [94]. Therefore, to better characterization of Ub chains by TDP, it’s important to optimize the instrumental parameters [95]. Lee et al. used a TDP strategy that utilized electron-transfer/collision-induced dissociation (ETciD) activation to achieve extensive fragmentation to facilitate the characterization of chain topography and lysine linkage sites, such as K48 linkage and K63 linkage [96]. Thus, while TDP has been used to analyze the topologies of some well-defined ubiquitylated proteins, the application of TDP in the analysis of complex, heterogenous mixtures has not been realized. With advantages in sample preparation and analytical approaches, TDP will play a vital role in analyzing the topologies of Ub chains for a complex system.

MS-based proteomics plays an important role in the identification of Ub chains and linkages. However, it should be noted that strategies for detecting branched chains by MS usually include an enrichment step to increase the recovery of substrates before digestion or direct analysis.

## Prediction of protein ubiquitination via computational algorithms

The current methodologies for systematically analyzing protein ubiquitination can be divided into two categories: MS-based strategies and computational strategies. MS-based experimental strategies are often expensive, labor-intensive, and time-consuming. Compared with MS-based strategies, prediction protein ubiquitination using a variety of machine-learning methods can provide simple and rapid research solutions, and provide valuable information for further laboratory studies [97].

Over the past decade, researchers have achieved great success in applying different feature extraction methods to predict protein ubiquitination sites, such as machine-learning algorithms. These computational approaches predict new ubiquitination sites by learning sequence context characteristics of ubiquitination sites of the experimentally verified ubiquitination sites. UbiPred was the first tool reported by Tung et al. for predicting ubiquitination sites, which was implemented by using a Support Vector Machine (SVM) with 31 informative physicochemical features selected from published amino acid indices [98, 99]. The authors utilized UbiPred and identified 23 ubiquitylation sites, which were further validated. In 2018, He et al. reported a multimodal deep architecture to identify the ubiquitination sites and evaluated their method on the available database PLMD, leading to 66.4% specificity, 66.7% sensitivity, and 66.43% accuracy [100]. Recently, Wang et al. reported a new method, named HUbipPred, which utilized the binary encoding and physicochemical properties of amino acids as training input and integrated two kinds of neural networks to build the model [101]. HUbiPred greatly improved the prediction accuracy compared to previous predictors such as DeepUbi and hCKSAAP_UbSite. At present, more and more bioinformatics tools have been developed for predicting protein ubiquitination sites. We refer the readers to a series of excellent reviews discussing the different methods, predictive algorithms, functionality, and properties for predicting protein ubiquitination [97, 102, 103].

Since there is no universal algorithm that can accurately predict protein ubiquitination sites, the fusion of multiple computational methods may be an effective method to comprehensively predict protein ubiquitination sites. In addition, a large amount of protein ubiquitination data has been rapidly accumulated in the last decade. As expected, the improvement of the ubiquitination sequence logo is consistent with the increased sensitivity of the recently developed predictor [103]. Because computational methodology always introduces false-positive results, verification of the prediction results through experimental methods is required.

## Conclusions

Protein ubiquitination is one of the most difficult PTMs to be identified due to its large size, low abundance, and dynamic regulation. To identify protein ubiquitination, a diversity of enrichment approaches to ubiquitination at multiple levels have been developed, including the protein level and the peptide level (Table 1). Since MS analysis has become the most powerful tool to precisely identify PTMs, the developed enrichment approaches combined with the advanced MS enabled the identification of tens of thousands of ubiquitination sites corresponding to thousands of ubiquitinated proteins. Considering the fact that MS-based experimental methods are often expensive, labor-intensive, and time-consuming, bioinformatics approaches and tools based on machine learning from the reported ubiquitination dataset have recently been developed for predicting protein ubiquitination sites. However, these tools are constructed based on different training libraries, prediction algorithms, functionality, and features, complicating their utilities and applications. Despite various limitations, it is now possible for researchers to analyze thousands of ubiquitination sites using experimental methods or predicting approaches. However, the prediction of ubiquitination sites by machine learning underlies the complex nature of Ub chain topologies. Because this approach only predicts the ubiquitination sites rather than the topologies of Ub chains, experimental methods are the only way to get insights into the architecture of Ub chains.

**Table 1:** Comparison of different strategies for ubiquitination characterization: from ubiquitinated protein to ubiquitin chain architecture

Levels	Approaches	Advantages	Disadvantages	Application	Refs
At the protein level	Ub tagging-based approaches	• Can remove the majority of non-ubiquitinated proteins • Can identify ubiquitination sites and localize them to proteins	• Require expressing ubiquitin tag which may behave differently from endogenous ubiquitin and impair the identification accuracy of ubiquitylation • Low efficiency for ubiquitylation identification • Limit its application in tissues	Screen and validation of ubiquitinated substrates in cells	[34–36]
	Ub antibody-based approaches	• Can purify endogenous ubiquitinated proteins • Enrich the linkage specific ubiquitylated proteins by linkage-specific antibodies • Application in all samples	• High cost of antibodies • High background derived from binding proteins • Low efficiency for ubiquitylation identification	Validation of ubiquitinated substrates and their linkage types in all samples	[37–40]
	UBD-based approaches	• Do not require expressing ubiquitin tag and antibodies • Can purify endogenous ubiquitinated proteins • Enrich the linkage specific ubiquitylated proteins by linkage-specific UBDs	• lower affinity of monoubiquitylated proteins • Low efficiency for ubiquitylation identification • High background derived from UBAs and UBDs	Screen of ubiquitinated proteins and their linkage types in all samples	[47–55]
At the peptide level	Anti-diGly antibody-based approach	• Can identify large number of ubiquitination sites • High efficiency for ubiquitination identification	• High cost of antibody • False positive identification generated from ISG15 and NEDD8 modification • Cannot identify N-terminal ubiquitylation sites • Cannot reveal any information on ubiquitin chain topology	Profiling of ubiquitination sites in all samples	[57–65]
	UbiSite antibody-based approach	• Can identify large number of ubiquitination sites with high efficiency • Can identify N-terminal ubiquitination sites • Can avoid the interference of ISG15 and NEDD8 modification	• High cost of antibody • The longer ubiquitin remnants on the ubiquitination sites hamper the identification of the ubiquitinated peptides • Cannot reveal the information on ubiquitin chain topology	Profiling of ubiquitination sites in all samples	[70]
	Antibody-free approaches	• Do not require antibody • Low cost • Can identify large number of ubiquitination sites	• Low through-put compared with antibody-based approaches • Artifacts derived from ubiquitin mutation and chemical derivatization	Screen of ubiquitination sites in cells	[75–77]
At the topology level	Bottom-up proteomics, e.g. Ub-AQUA and UbiCRest	• Can dissect the ubiquitin chain architecture	• Cannot well distinguish branched from mixed ubiquitin chains • Low specificity in identifying linkage types • Cannot effectively analyze the heterotypic chains	Validation of the ubiquitin chain linkage in all samples	[79–86]
	Middle-down proteomics, e.g. Ub-clipping	• Can dissect the branched ubiquitin chains • Can reveal the ratio of branched to unbranched linkages	• Cannot dissect the chain linkage types at the branched point	Screen and validation of ubiquitination sites and its topologies in all samples	[91–96]
	Top-down proteomics	• Can dissect the branched ubiquitin chains • Can reveal the ratio of branched to unbranched linkages • Can dissect the chain linkage types at the branched point	• Low signal-to-noise (S/N) fragments with increasing molecular weight • Lack in in sample preparation and analytical approaches	Application in identifying ubiquitination sites and its topologies in all samples	[28, 97–99]

The complexity of ubiquitination stems from the ability to form the polymerization with different length (number of Ub molecules), linkage, and overall architecture. Conformation of the polyUb plays a vital role in regulating the function of substrates in diverse physiological and pathological processes. Although a series of techniques have been developed to detect branched Ub chains, none of them can reveal the topology and length of branched chains. Methodology breakthrough is desperately needed to provide systematic insights into the overall architecture of Ub chain, such as the exact structure of different linkages. Top-down proteomics (TDP) may be a promising tool to map the structure of Ub chains bearing different lengths, linkages, and architectures. However, how to solve the S/N ratio of the ubiquitinated substrates is a great challenge.

In summary, protein ubiquitination analysis has made significant progress over the past decade. However, significant challenges remain in this area. Promising methods and dedicated databases will help us untangle the complexities of ubiquitination and facilitate the discovery of biomarkers associated with abnormal protein ubiquitination in a variety of diseases.

## Data Availability

Not applicable.

## Abbreviations

PTM: Post-translational modification
Ub: Ubiquitin
DUBs: Deubiquitinases
M: Methionine
K: Lysine
R: Arginine
UBDs: Ubiquitin binding domains
LT: Large tumor
MS: Mass spectrometry
TUBEs: Tandem-repeated Ub-binding entities
TR-TUBEs: Trypsin-resistant tandem-repeated Ub-binding entities
ThUBD: Tandem hybrid ubiquitin binding domains
AQUA: Absolute quantification
diGly: Diglycine
LFASP: Large-scale filter-aided sample preparation
DIA: Data independent acquisition
IAA: Iodoacetamide
COFRADIC: COmbined FRActional DIagonal Chromatography
USP2cc: USP2 catalytic core domain
BOC: Tert-butyloxycarbonyl
StUbEx: Stable Tagged Ub Exchange
BUP: Bottom-up proteomics
UbiCRest: Ubiquitin chain restriction
MDP: Middle-down proteomics
TDP: Top-down proteomics
S/N: Signal-to-noise
